# Small B-cell lymphoma highly suspicious for splenic marginal zone lymphoma with *MYD88* L265P mutation and *MYC* copy-number gain presenting as severe autoimmune hemolytic anemia: a case report with literature review

**DOI:** 10.3389/fimmu.2026.1767267

**Published:** 2026-02-11

**Authors:** Xiaoyan Liu, Guoyang Zhang, Liping Ma

**Affiliations:** Department of Hematology, Sun Yat-sen Memorial Hospital, Sun Yat-sen University, Guangzhou, China

**Keywords:** autoimmune hemolytic anemia, MYC amplification, MYD88 L265P, small B-cell lymphoma, splenic marginal zone lymphoma

## Abstract

In indolent small B-cell lymphomas, autoimmune cytopenia and histologic transformation pose important diagnostic and management challenges. *MYD88* and *MYC* abnormalities are rarely reported in splenic marginal zone lymphoma (SMZL), and their role in immune dysregulation and transformation remains unclear. We describe a 44-year-old man with a small B-cell lymphoma highly suspicious for SMZL, who presented with severe warm autoimmune hemolytic anemia, massive splenomegaly, and lymphocytosis. He harbored both *MYD88* L265P mutation and *MYC* copy-number gain within a complex karyotype, in the absence of splenic histology. This case, together with a review of the literature, suggests that such “clinically indolent but genetically high-risk” SMZL-like lymphomas may carry a non-negligible risk of progression and transformation and therefore require close longitudinal monitoring. Further accumulation of similar cases and molecular data is needed to clarify the prognostic and biological impact of *MYD88* and *MYC* alterations in this setting.

## 1 Introduction

Splenic marginal zone lymphoma (SMZL) is an indolent non-Hodgkin’s lymphoma that originates from post-germinal center memory B-cells. It is characterized by splenomegaly, with or without peripheral blood lymphocytosis and bone marrow involvement by small clonal B-cells (1). It accounts for approximately 2% of all lymphomas and 20% of marginal zone lymphomas. The median overall survival is 8–10 years, but a subset of patients may experience histologic transformation to diffuse large B-cell lymphoma (DLBCL) or other aggressive lymphomas, which is associated with poor outcome (2). Clinically, SMZL complicated by autoimmune hemolytic anemia (AIHA) is relatively uncommon and can be easily misdiagnosed as primary AIHA or other hematological diseases. Both *MYD88* L265P mutation and *MYC* aberrations are uncommon in SMZL. Notably, the latter is a well-established marker of high-grade disease and poor prognosis in B-cell lymphomas (3, 4). Here, we report a patient who initially presented with peripheral blood lymphocytosis and was later readmitted with severe anemia due to worsening warm AIHA. The bone marrow pathology and immunohistochemistry indicated a small B-cell lymphoma. Then combined with the clinical features, it was highly suspected to be SMZL. *MYD88* L265P mutation and *MYC* gene amplification together with other complex cytogenetic abnormalities were detected. In the absence of splenic pathology as the diagnostic gold standard, we classified this case as “a small B-cell lymphoma with high-risk molecular features and highly suspected SMZL”. Through this case description and a brief review of the literature, we focus on the clinical and molecular characteristics of small B-cell lymphomas with high-risk genetic abnormalities. We also discuss risk assessment and therapeutic strategies in situations where the diagnosis cannot be fully established.

## 2 Case description

A 44-year-old man was first admitted to the rheumatology department of our hospital on 25 June 2024 with a one-year history of recurrent polyarticular redness, swelling and pain. He was diagnosed with chronic gouty arthritis. Complete blood count (CBC) showed a white blood cell (WBC) of 33.64×10^9^/L, hemoglobin (Hb) 116 g/L, platelet count 239×10^9^/L, and 66% atypical lymphocytes in the peripheral blood. Physical examination showed splenomegaly. Bone marrow biopsy at that time demonstrated nodular aggregates of small lymphoid cells with a Ki-67 index of approximately 40%, consistent with a small B-cell lymphoma, but the exact subtype could not be determined. PET-CT and systemic therapy were not pursued, and the patient was discharged without specific lymphoma-directed treatment.

On 19 February 2025, he presented again, this time to the hematology department, with a two-week history of exertional dyspnea and progressive jaundice. On examination, the heart rate was 86 beats/min with a regular rhythm, and the spleen was markedly enlarged (massive splenomegaly), firm in consistency, with the lower pole extending beyond the level of the umbilicus. No other distinct positive physical signs were identified.

Laboratory tests showed: WBC 19.18×10^9^/L, Hb 54 g/L, platelet count 125×10^9^/L, with atypical lymphocytes accounting for 58% of leukocytes; total bilirubin 38.5 μmol/L, indirect bilirubin 29.7 μmol/L, uric acid 659 μmol/L, lactate dehydrogenase (LDH) 498 U/L, and ferritin 436.0 μg/L; haptoglobin was < 0.0543 g/L. The direct antiglobulin test (Coombs) was positive, as was the indirect antibody screening test. Urinalysis showed 1+ occult blood, 1+ protein, and elevated urobilinogen at 66.0 μmol/L (**Table 1**). Serologic tests for hepatitis B and C, syphilis, HIV, Epstein–Barr virus DNA, cytomegalovirus DNA, folate, vitamin B12, erythropoietin, thyroid function, and antinuclear antibody were all within normal limits or negative. Immunofixation electrophoresis was negative for monoclonal immunoglobulins. Whole-body PET-CT demonstrated diffusely increased radiotracer uptake in the bone marrow cavity of the axial skeleton and proximal segments of the long bones, most prominent in the pelvic bones (SUVmax ≈ 6.4), without apparent cortical bone destruction. The spleen was massively enlarged (anteroposterior diameter equivalent to ~9 rib units, thickness ~88 mm, inferior pole extending down to the L4 vertebral level) with homogeneous, moderately increased uptake (SUVmax 4.8). No hypermetabolic lymphadenopathy or extranodal masses were evident.

**Table 1:** Initial laboratory findings of the patient.

Parameter	Value	Reference range	Unit
Hematology			
White Blood Cells (WBC)	19.18	3.5–9.5	×10^9^/L
Hemoglobin (Hb)	54	130–175	×10^9^/L
Platelet Count	125	125–350	×10^9^/L
atypical lymphocytes	58%	–	–
Biochemistry			
Total Bilirubin	38.5	0–23	μmol/L
Indirect Bilirubin	29.7	3.4–18.8	μmol/L
Uric acid	659	208–428	μmol/L
Lactate Dehydrogenase (LDH)	498	108–25	U/L
Ferritin	436	20–300	μg/L
Haptoglobin	<3.0	0.3–2	g/L
Immunology			
Direct Antiglobulin Test	Positive	Negative	–
Immunofixation electrophoresis	Negative	Negative	–

**Figure 1:**
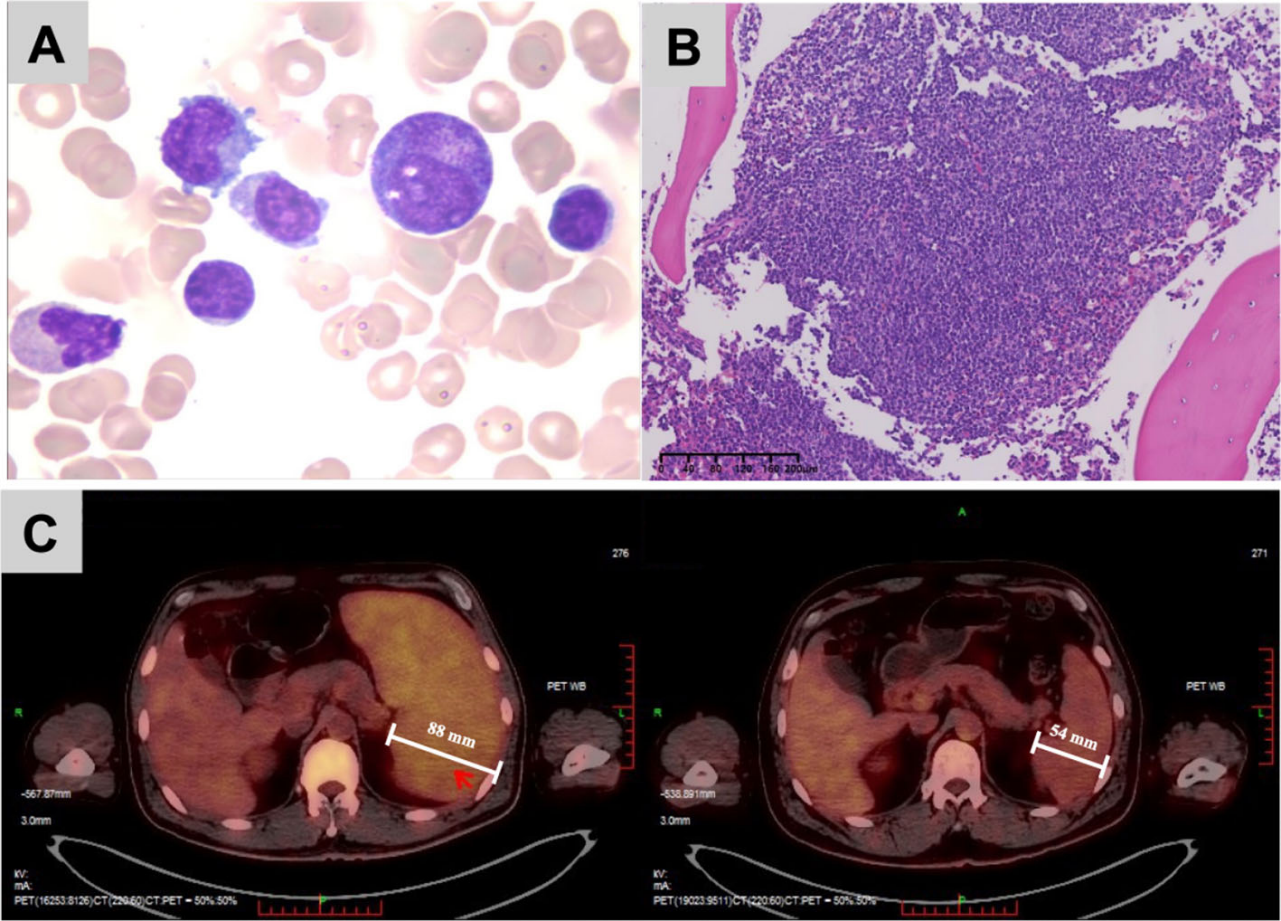
Bone marrow morphology and PET-CT findings at diagnosis and after treatment. **(A)** Bone marrow smear showing a population of mature small lymphocytes with scant cytoplasm and occasional cytoplasmic projections (Wright–Giemsa stain, ×1000). **(B)** Bone marrow biopsy demonstrating nodular and interstitial infiltrates of small B lymphocytes consistent with a small B-cell lymphoma in the marginal zone spectrum (H&E stain, ×10). **(C)** The PET-CT images of the spleen at diagnosis (left) and after six cycles of R-CVP (right). At baseline, the spleen is markedly enlarged (maximal thickness approximately 88 mm; SUVmax 4.8). On follow-up, splenic size is clearly reduced (thickness approximately 54 mm; SUVmax 2.9), with a corresponding decrease in metabolic activity.

Bone marrow morphology showed lymphocytosis, and the cells were predominantly small, round to oval, with scant grey-blue to deep blue cytoplasm; some exhibited cytoplasmic projections (**Figure 1A**). The flow cytometry identified 29.98% CD5^-^CD10^-^ small monoclonal B lymphocytes. Bone marrow biopsy histology: small lymphoid cells can be seen in clusters or sheets in the bone marrow (**Figure 1B**); the immunohistochemistry showed: CD3 (−), CD5 (−), CD10 (−), CyclinD1 (−), LEF-1 (−), CD56 (−), CD20 (+), CD79a (+), Mum-1 (focally +), CD21 (focally +), Bcl-2 (~80%+), Bcl-6 (~15%+), C-Myc (~5%+), CD138 (focally +), kappa (focally +), lambda (−), CD34 (rare +), and TdT (rare +). The Ki-67 proliferation index was approximately 40%. Thus, the histologic and immunohistochemical features supported a diagnosis of small B-cell lymphoma, favoring marginal zone B-cell lymphoma. The chromosome karyotype showed a complex abnormal clone: 49, XY, 8q-, +18, +mar×2[7]/49, idem,14q- [2]/46, XY [1]. Fluorescence *in situ* hybridization (FISH) did not detect *MYC-IGH* fusion, but a *MYC* probe detected copy number gain/amplification in approximately 10% of nuclei with occasional break-apart signals; *TP53* deletion was not detected. Targeted next-generation sequencing (NGS) identified a *MYD88* p.L265P (c.794T>C) mutation with a variant allele frequency of 22.4%. Multiple chromosomal/segmental copy number alterations were also detected, including gains of 3q, 8q24.11–q24.21, gains of chromosome 18, deletions of 16p and copy-neutral loss of heterozygosity of 17q.

**Figure 2:**
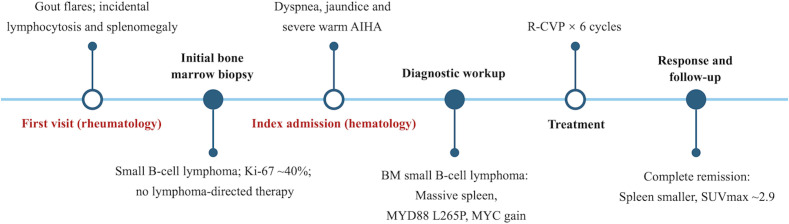
Timeline of diagnostic events and treatments of SMZL; R-CVP: rituximab, cyclophosphamide, vincristine, and prednisone.

The final diagnosis was a small B-cell lymphoma highly suspicious for SMZL (stage IV with B symptoms), with concomitant *MYD88* L265P mutation and *MYC* amplification, complicated by warm AIHA. In the absence of splenic histopathology, we could not exclude the possibility of occult DLBCL-like transformation within the spleen. Upon admission to the hematology department and after completion of the diagnostic workup confirming warm AIHA, systemic corticosteroid therapy with intravenous methylprednisolone was initiated. It was continued concomitantly with the first cycle of R-CVP immunochemotherapy (rituximab, cyclophosphamide, vincristine, and prednisone), followed by a gradual taper according to clinical response and hemolysis parameters. In total, the patient was treated with six cycles R-CVP regimen. The timeline of the diagnostic events and the treatments used during the whole period was as follows (**Figure 2**). After the first cycle, his hemoglobin and bilirubin levels returned to the normal range, indicating effective control of both lymphoma and AIHA. Following completion of six cycles, repeat PET-CT showed a marked reduction in splenic size (thickness ~54 mm, SUVmax 2.9, **Figure 1C**) and decreased diffuse bone marrow uptake, consistent with complete remission. The patient remains in clinical remission under regular follow-up. He has now been followed for 5 months after the end of treatment, during which he has remained asymptomatic with stable hemoglobin and no biochemical evidence of recurrent hemolysis, consistent with an ongoing complete clinical remission.

## 3 Discussion

In this report, we describe a middle-aged man with a small B-cell lymphoma highly suspicious for SMZL, who presented with massive splenomegaly, marked lymphocytosis and severe warm AIHA. He also carried with both *MYD88* L265P and *MYC* copy-number gain within a complex karyotype. This combination of prominent AIHA, “clinically indolent but genetically high-risk” disease and the lack of splenic histology raises concern for occult or future histologic transformation and illustrates several diagnostic and therapeutic challenges.

### 3.1 AIHA and SMZL

AIHA is caused by immune dysregulation leading to the production of autoantibodies, which in turn accelerate the destruction of red blood cells (hemolysis). This exceeds the compensatory capacity of the bone marrow and ultimately leads to anemia (5). AIHA can be primary or secondary; and approximately 50% of secondary AIHA cases are associated with autoimmune diseases or lymphoproliferative disorders (6). Among these, chronic lymphocytic leukemia (CLL) is most frequently associated with AIHA, whereas the link with other lymphoma subtypes has been less commonly reported (7).The pathophysiological mechanisms of lymphoma-associated AIHA remain incompletely understood. Studies in CLL have suggested that immune disorders (including both humoral and cellular immune abnormalities), unmutated *IGHV* status, specific *IGHV* frameworks, and adverse cytogenetic lesions (such as del(17p) and/or del(11q)) are risk factors for AIHA (8). Thieblemont et al. (9) retrospectively analyzed 81 patients with SMZL and found that 16 cases experienced autoimmune events (10 cases of AIHA, 4 cases of immune thrombocytopenia, and 2 cases of acquired coagulation disorders). Other studies have suggested that such autoimmune events may be manifestations of the active stage of SMZL (10). Among the reported cases of MZL complicated by AIHA, some patients were diagnosed with MZL shortly after the onset of AIHA, some developed AIHA after an established lymphoma diagnosis, and others were diagnosed with MZL several years after the initial AIHA episode. Overall, when lymphoma, especially aggressive subtypes, is complicated by AIHA, the clinical course is often severe and may present with severe or even life-threatening anemia. Refractory AIHA is also common in this setting. These patients often respond poorly to standard immunosuppressive therapy, and corticosteroid use may mask lymphoma-related manifestations, contributing to diagnostic delay. Therefore, AIHA patients with concomitant hepatosplenomegaly, lymphadenopathy, or recent unintentional weight loss should be promptly evaluated for underlying lymphoma. Currently, AIHA is considered both a potential adverse prognostic factor and a marker of disease activity in SMZL (11).

In our patient, lymphocytosis and splenomegaly were already evident at the initial rheumatology visit for gout, but no further hematologic work-up was undertaken. Eight months later, severe warm AIHA with profound anemia and jaundice was the main reason for admission. If management had focused solely on “primary AIHA,” the lymphoma might have remained undiagnosed. Instead, the combination of AIHA, massive splenomegaly and lymphocytosis prompted bone marrow evaluation, which revealed a clonal small B-cell lymphoma. The rapid normalization of hemoglobin and bilirubin after the first cycle of R-CVP underscores that effective control of the underlying lymphoma is crucial for resolving lymphoma-associated AIHA. This case highlights the importance of evaluating AIHA patients with splenomegaly, lymphocytosis, or other “red flags” for an underlying small B-cell lymphoma. Conversely, it also underscores that autoimmune cytopenias may serve as a marker of disease activity in SMZL.

Notably, this patient had a long history of chronic gouty arthritis before the diagnosis of lymphoma, suggesting a potential pathogenetic link rather than simple coexistence. Numerous studies have demonstrated that patients with autoimmune diseases such as rheumatoid arthritis have a significantly increased risk of non-Hodgkin lymphoma (12). This risk appears to correlate with disease activity and cumulative inflammatory burden. Together, these findings support the hypothesis that chronic inflammation can drive B-cell clonal expansion and lymphomagenesis. Although gouty arthritis is not a classic autoimmune arthritis, monosodium urate crystals can activate the NLRP3 inflammasome and drive IL-1β mediated inflammation (13). The resulting chronic, low-grade inflammatory milieu may favor lymphomagenesis, although a direct causal link between gout and lymphoma has not been established. Therefore, the pre-existing gouty arthritis in this case may have provided, at least in part, a permissive background for abnormal B-cell clonal proliferation and subsequent evolution. This raises the possibility that the sequential occurrence of these two conditions may not be entirely coincidental.

### 3.2 Significance of the *MYD88* L265P mutation in the SMZL

The *MYD88* L265P mutation is a common somatic point mutation in the *MYD88* gene (myeloid differentiation primary response 88; c.794T>C), leading to substitution of leucine by proline at codon 265 (p. Leu265Pro). This mutation constitutively activates NF-κB and JAK/STAT signaling, promoting B-cell proliferation and survival, and contributing to lymphomagenesis (14, 15). It is detected in > 90% of patients with lymphoplasmacytic lymphoma/Waldenström macroglobulinemia (LPL/WM) and is closely associated with IgM monoclonal gammopathy and plasmacytic differentiation (14, 16). In contrast, it is found only in a minority of SMZL cases, and is rarely seen in other marginal zone lymphoma subtypes, which may complicate differential diagnosis when present (14, 17–20). In a retrospective study, Azahara et al. (21) used TaqMan allele-specific PCR to assess 114 MZL patients (7 nodal MZL, 21 MALT lymphomas, and 86 SMZL) and 24 LPL patients. The *MYD88* L265P mutation was detected in 15% of SMZL cases (13/86) and in 79% of LPL cases (19/24), but was absent in nodal MZL and MALT lymphoma. Compared with *MYD88* wild-type cases, patients harboring the mutation were more likely to have an IgM monoclonal gammopathy (70% vs. 18%) and higher IgM levels (3.0–11.9 g/L vs. 3.7–6.6 g/L), whereas age, hemoglobin, LDH, β2-microglobulin, serum albumin, and prior hepatitis B virus infection did not differ significantly. Histologically, SMZL cases with *MYD88* L265P mutation more frequently exhibited plasmacytic differentiation (9/13, 69%).

In our case, bone marrow smears showed 39.5% mature small lymphocytes, some with cytoplasmic (villous) projections, in keeping with previously described cytologic features of SMZL (22). Bone marrow biopsy revealed nodular and interstitial infiltrates of small B-cells expressing CD20 and CD79a but negative for CD3, CD5, CD10 and CyclinD1, which supporting a diagnosis within the spectrum of MZL (23). Notably, the Ki-67 index of about 40% was higher than usually reported in classical indolent SMZL and suggests a more proliferative clone. Whole-body PET-CT showed diffuse, moderately increased FDG uptake in the spleen (SUVmax ~4.8). Previous studies have shown that the SUVmax of aggressive or transformed lymphomas is generally higher than that of indolent lymphomas. However, there is substantial overlap in SUV ranges between these entities, and a single SUV threshold cannot reliably distinguish indolent from aggressive disease (24). Targeted NGS detected the *MYD88* L265P mutation with a variant allele frequency of 22.4%, indicating a sizable clonal population. However, the patient had no IgM paraproteinemia or hyperviscosity, no cold agglutinin disease or cryoglobulinemia, and no peripheral or central nervous system involvement. Bone marrow morphology did not show typical lymphoplasmacytic differentiation, and CD138 and Mum-1 were only focally positive. Taken together, these findings effectively excluded LPL/WM and favored classification as a small B-cell lymphoma highly suspicious for SMZL. From an immunologic perspective, *MYD88*-driven signaling may help sustain both lymphoma cell survival (14) and a pro-inflammatory cytokine milieu that favors autoantibody production, potentially contributing to autoimmune cytopenias (25) such as AIHA. However, this hypothesis still requires validation.

### 3.3 Cytogenetic abnormalities in SMZL

Copy number alterations and complex karyotypes are key features of many B-cell neoplasms. In MZL, trisomies of chromosomes 3 and 18 and mutations in chromatin-remodeling genes are relatively common (26). Salido et al. (27) analyzed karyotypes from 330 SMZL patients and found that 53% had complex karyotypes (≥3 abnormalities), with frequent lesions involving gains of 3/3q and 12q, deletions of 7q and 6q, and translocations affecting 8q/1q/14q. Among these, 7q deletion (14–44%) and 3q gains (20–30%) are particularly characteristic of SMZL. Our patient’s karyotype showed multiple structural and numerical abnormalities, including 8q deletion, 14q deletion and trisomy 18. FISH did not detect a classic *MYC-IGH* fusion, but *MYC* copy number gain was observed in approximately 10% of nuclei. NGS confirmed gains in 8q24.11-q24.21 encompassing the *MYC* locus, along with gains of chromosome 18. In addition, NGS revealed cryptic abnormalities not captured by conventional karyotyping or locus-specific FISH, including 3q gains, 16p deletions, and copy-neutral loss of heterozygosity on 17q. Among these, gains of 3q and trisomy 18 are recurrent abnormalities in SMZL, while 17q may suggest potential deregulation of the *TP53* pathway. Importantly, this case exhibited *MYC* copy number gain.

*MYC* is a multifunctional transcription factor that promotes tumorigenesis by enhancing cell proliferation, stimulating angiogenesis, and increasing genomic instability (28). In lymphomas, *MYC* abnormalities predominantly manifest as gene rearrangements and amplifications, whereas point mutations are relatively rare. It is crucial to distinguish *MYC* copy number gain/amplification from *MYC* rearrangement, as their biological and prognostic implications may differ and remain incompletely defined. In DLBCL, *MYC* amplification has been associated with adverse prognosis (29). In mantle cell lymphoma, *MYC* overexpression is an independent predictor of poor outcome, and both amplification and translocation events involving *MYC* may contribute to disease progression (30). Although *MYC* abnormalities are exceedingly rare in MZL, studies of MZL undergoing histologic transformation to DLBCL have shown marked upregulation of MYC protein. Even in the absence of detectable *MYC* rearrangement, this upregulation can contribute to disease progression and aggressive transformation (31). Therefore, *MYC* alterations are often regarded as a “molecular warning signal” of progression or impending transformation. Rearrangements often suggest highly aggressive disease. Even copy-number gains may signal clonal evolution and justify close surveillance for increasing disease burden, a higher Ki-67 index, or morphologic transformation (e.g. to DLBCL or Burkitt-like lymphoma).

Although a classic *MYC* translocation was not identified in this patient, the presence of *MYC* amplification together with 3q gains, trisomy 18 and other copy number aberrations indicates considerable genomic complexity and potential adverse biological behavior. Clinically, this patient should therefore be considered part of a high-risk subset of SMZL-like lymphomas with a non-negligible risk of histologic transformation, warranting closer follow-up and periodic re-evaluation.

### 3.4 Diagnostic limitations, transformation risk, and clinical implications

The major limitation in this case is the absence of splenic histopathology, which represents the gold standard for diagnosis and grading of SMZL. Our conclusions rely on an integrated assessment of bone marrow histology, immunohistochemistry, cytogenetics, molecular findings, and clinical features. Moderately increased splenic FDG uptake (SUVmax ~4.8), a relatively high Ki-67 index (~40%), and *MYC* copy number gain are uncommon in prototypical indolent SMZL and suggest a degree of biological aggressiveness. Therefore, focal transformation to DLBCL within the spleen cannot be completely excluded. At the same time, the eight-month interval between the initial detection of lymphocytosis and the definitive diagnosis was characterized by relatively slow clinical progression. The patient then achieved complete remission after six cycles of rituximab-based immunochemotherapy, which is consistent with an indolent clinical course. Integrating these observations, we consider the most appropriate designation to be a “small B-cell lymphoma highly suspicious for SMZL with high-risk molecular features,” and explicitly acknowledge the residual diagnostic uncertainty.

This case underscores that, in real-world settings where splenic tissue is not available, multidimensional laboratory tests (including pathology, flow cytometric immunophenotyping, cytogenetics, and NGS) are essential for the precise classification of small B-cell lymphomas and for assessing the risk of histologic transformation. In our patient, five months of post-treatment follow-up have documented a sustained complete remission without recurrent hemolysis, although longer observation is needed to clarify the long-term course. Once signals of “disease progression” are observed, the treatment should be promptly adjusted, and targeted pathway drugs (such as BTK inhibitors targeting the *MYD88* pathway), clinical trials should be actively considered should be considered for inclusion.

## Data Availability

The original contributions presented in the study are included in the article/supplementary material. Further inquiries can be directed to the corresponding author.
